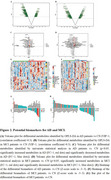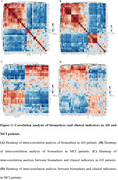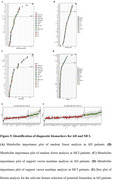# Metabolomics and lipidomics study on serum metabolite signatures in Alzheimer's disease and mild cognitive impairment

**DOI:** 10.1002/alz70856_104134

**Published:** 2025-12-26

**Authors:** Yingren Mai, Zhiyu Cao, Qun Yu, Jun Liu

**Affiliations:** ^1^ The Second Affiliated Hospital of Guangzhou Medical University, Guangzhou, Guangdong, China; ^2^ Department of Neurology, the Second Affiliated Hospital of Guangzhou Medical University, Institute of Neuroscience, the Second Affiliated Hospital of Guangzhou Medical University, Guangzhou, Guangdong, China

## Abstract

**Background:**

Alzheimer's disease (AD) and mild cognitive impairment (MCI) are leading causes of dementia in the elderly worldwide, characterized by abnormal cognition and behavior, which complicates diagnosis and treatment. Metabolites play a critical role in cellular process related to the pathogenesis of AD and MCI. However, the metabolic alterations, particularly in lipid metabolism, in AD and MCI are poorly understood.

**Method:**

In this study, we applied a quantitative and targeted metabolomics approach to a cohort of AD patients (*n* = 22), MCI patients (*n* = 19) and cognitively normal (CN) (*n* = 19) using ultra‐performance liquid chromatography triple quadrupole mass spectrometry to identify metabolic changes associated with AD and MCI.

**Result:**

Compared to CN, we identified 32 differential metabolites in AD and 49 in MCI serum. Notably, differential metabolites related to AA, organic acid, FA, phosphatidylcholine (PC), sphingomyelin (SM) metabolism in AD and free fatty acid (FFA), acylcarnitine, PC, SM in MCI were strongly associated with cognitive level, memory, attention and execution function as evaluated by scales including Mini‐Mental State Examination (MMSE), the Alzheimer's Disease Assessment Scale‐cognitive Section (ADAS), the Montreal Cognitive Assessment (MoCA), the Clinical Dementia Rating (CDR), the Auditory Verbal Learning Test (AVLT) and the Trail Making Test (TMT). Pathway analysis based on the differential metabolites revealed perturbation in pathways related to phospholipid metabolism, sphingolipid metabolism, amino acids (AAs) metabolism, beta oxidation of FAs, and carnitine metabolism. Using random forest (RF), support vector machine (SVM) and Boruta analysis for classification and validated by gradient boosting (GB), logistic regression (LR) and random forest diagnostic model, we identified panels of 10 metabolites in AD and 13 metabolites in MCI that effectively discriminate AD and MCI individuals from CN with high accuracy, sensitivity and specificity.

**Conclusion:**

In summary, this novel study combing metabolomics and lipidomics approaches and found that perturbations in serum sphignolipids, glycerophospholipids, AAs, FFA and acylcarnitines are consistently associated with pathology and progression of AD and MCI. These metabolic biomarkers provided promising molecular targets for the early diagnosis and treatment of AD and MCI.